# Detection of EGFR Mutations by TaqMan Mutation Detection Assays Powered by Competitive Allele-Specific TaqMan PCR Technology

**DOI:** 10.1155/2013/385087

**Published:** 2013-12-01

**Authors:** Cristin Roma, Claudia Esposito, Anna Maria Rachiglio, Raffaella Pasquale, Alessia Iannaccone, Nicoletta Chicchinelli, Renato Franco, Rita Mancini, Salvatore Pisconti, Antonella De Luca, Gerardo Botti, Alessandro Morabito, Nicola Normanno

**Affiliations:** ^1^Laboratory of Pharmacogenomics, Centro di Ricerche Oncologiche di Mercogliano (CROM), Istituto Nazionale per lo Studio e la Cura dei Tumori “Fondazione Giovanni Pascale”(IRCCS), 83013 Mercogliano, Italy; ^2^Cell Biology and Biotherapy Unit, Istituto Nazionale per lo Studio e la Cura dei Tumori “Fondazione Giovanni Pascale”(IRCCS), 80131 Naples, Italy; ^3^Surgical Pathology Unit, Istituto Nazionale per lo Studio e la Cura dei Tumori “Fondazione Giovanni Pascale”(IRCCS), 80131 Naples, Italy; ^4^Department of Molecular and Clinical Medicine, Laboratory of Molecular and Cellular Biology, Università “La Sapienza”, 00189 Rome, Italy; ^5^Department of Surgery “A. Valdoni”, Laboratory of Molecular and Cellular Biology, Università “La Sapienza”, 00161 Rome, Italy; ^6^Medical Oncology Unit, SS Annunziata Hospital, 74123 Taranto, Italy; ^7^Medical Oncology Unit, Department of Thoracic Surgical and Medical Oncology, Istituto Nazionale per lo Studio e la Cura dei Tumori “Fondazione Giovanni Pascale”(IRCCS), 80131 Naples, Italy

## Abstract

Epidermal growth factor receptor (EGFR) mutations in non-small-cell lung cancer (NSCLC) are predictive of response to treatment with tyrosine kinase inhibitors. Competitive Allele-Specific TaqMan PCR (castPCR) is a highly sensitive and specific technology. EGFR mutations were assessed by TaqMan Mutation Detection Assays (TMDA) based on castPCR technology in 64 tumor samples: a training set of 30 NSCLC and 6 colorectal carcinoma (CRC) samples and a validation set of 28 NSCLC cases. The sensitivity and specificity of this method were compared with routine diagnostic techniques including direct sequencing and the EGFR Therascreen RGQ kit. Analysis of the training set allowed the identification of the threshold value for data analysis (0.2); the maximum cycle threshold (Ct = 37); and the cut-off ΔCt value (7) for the EGFR TMDA. By using these parameters, castPCR technology identified both training and validation set EGFR mutations with similar frequency as compared with the Therascreen kit. Sequencing detected rare mutations that are not identified by either castPCR or Therascreen, but in samples with low tumor cell content it failed to detect common mutations that were revealed by real-time PCR based methods. In conclusion, our data suggest that castPCR is highly sensitive and specific to detect EGFR mutations in NSCLC clinical samples.

## 1. Introduction

The discovery of driver mutations in key genes involved in regulating proliferation and survival of cancer cells and the development of drugs capable to block such oncogenic mechanisms are leading to remarkable successes in translational medicine [1, 2]. However, the novel therapeutic approaches based on drugs directed against specific molecular agents are suitable only for molecularly selected populations of patients [3]. Therefore, molecular characterization is mandatory to identify patients which would most likely benefit from treatment with targeted therapies.

Mutations in the epidermal growth factor receptor (EGFR) gene in non-small-cell lung cancer (NSCLC) are predictive of response to treatment with tyrosine kinase inhibitors (TKIs) [4, 5]. These mutations are usually found in exons 18 through 21 of the TK domain of the EGFR and are either point mutations or in-frame small deletions or insertions [6]. Although more than 250 mutations of the EGFR have been described up to now, two variants, a single point mutation in exon 21, the L858R, and a series of small in-frame deletions in exon 19, account for approximately 90% of all EGFR mutations [6]. In order to determine whether an EGFR TKI or chemotherapy is the appropriate first-line therapy, guidelines recommend mutation testing for all patients with advanced NSCLC tumor and adenocarcinoma histology [7].

The sensitivity of assays for hot-spot mutation detection is a key issue in molecular diagnostics due to several limitations of tumor samples: the poor quality of the DNA extracted from formalin fixed paraffin embedded (FFPE) tissues, the low quantity of DNA available, and the contamination of tumor sample by nonneoplastic cells carrying wild type alleles [3]. Direct sequencing of PCR products is still considered the gold standard for the identification of mutations, but it is laborious and requires at least 40% to 50% of tumor cells content to prevent false negative results [7, 8]. The limited sensitivity of direct sequencing has created a need for alternative techniques to detect common mutations, such as well real-time PCR based assays, pyrosequencing, high resolution melting, and PNA-PCR clamp [9]. These new methods are faster and more sensitive than sequencing. For example, the real-time PCR based EGFR Therascreen RGQ kit, employing Scorpion probes and the ARMS technology, allows for selective amplification of mutated sequences leading to a sensitivity of 1% (Table 1).

Highly sensitive methods should be cautiously validated in routine diagnostic to ensure accuracy in tumor mutation testing. In this regard, Competitive Allele-Specific TaqMan PCR (castPCR) is a highly specific and sensitive technology, able to detect rare amounts of mutated DNA in a large background of normal, wild type genomic DNA [10]. An allele-specific primer and a FAM dye-labelled MGB (Minor Groove Binder) probe detect the mutant allele, while an MGB oligonucleotide blocker suppresses the wild type allele. Mutant allele assays are run with a gene reference assay that is designed to a mutation-free region of the gene. This approach is suitable for determining the presence or absence of a specific mutation in a sample with a high degree of specificity, enabling the detection of as little as 0.1% mutant allele in the presence of a wild type allele background (Table 1). In particular, sensibility for TaqMan Mutation Detection Assays (TMDA) has been described to be at least of 0.5% for most common EGFR mutations, including the L858R and exon 19 deletions [10]. However, the sensitivity of diagnostic tests is usually assessed by using limiting dilutions of recombinant DNA or genomic DNA derived from cell lines. Unfortunately, these experimental conditions do not resemble the clinical scenario, with particular regard to the analysis of DNA from FFPE tissue.

The aim of this study is to assess the feasibility of TaqMan Mutation Detection Assays (TMDA) based on castPCR technology to detect EGFR mutations in NSCLC clinical specimens and to compare this method with routine diagnostic techniques including direct sequencing and the EGFR Therascreen RGQ kit.

## 2. Materials and Methods

### 2.1. Samples

Archival tumor samples from 58 NSCLC and 6 colorectal carcinoma (CRC) patients were employed for this study. The NSCLC samples included 29 FFPE surgical specimens, 15 small FFPE biopsies obtained through fine needle aspirates, and 14 cytology smears.

The tumor cell content of each sample was assessed by experienced pathologists (RF and GB).

The following NSCLC cell lines obtained from ATCC (American Type Culture Collection) were used as controls: NCI-H1975 bearing both the L858R and T790M EGFR mutations and NCI-H1650 having the exon 19 E746_A750 deletion.

### 2.2. DNA Extraction

Genomic DNA (gDNA) was extracted using the QIAamp DNA Micro Kit (Qiagen) from cytological samples; the DNeasy Blood and Tissue Kit (Qiagen) from cancer cell lines; and the QIAmp DNA FFPE Tissue Kit (Qiagen) from FFPE tissues, according to manufacturer's instructions. Isolated gDNA was analyzed by 0.8% agarose gel electrophoresis to evaluate DNA quality. DNA quantity was assessed by using the NanoVue Spectrophotometer (GE Healthcare).

### 2.3. CastPCR

CastPCR was performed in 96-well plates preloaded with TaqMan Mutation Detection Assays, TaqMan EGFR Exon 19 Deletions Assay, and TaqMan Mutation Detection Reference Assays (Life Technologies) in 20 *μ*L reaction volume including 1x TaqMan genotyping master mix (Life Technologies), deionised water, and 10 ng DNA template. All the above mentioned assays have been developed by Life Technologies.

TaqMan Mutation Detection Assays were designed to detect the following EGFR mutations: c.2582T>A p.L861Q, c.2573T>G p.L858R, c.2156G>C p.G719A, c.2369C>T p.T790M, c.2303G>T p.S768I, c.2155G>A p.G719S, c.2155G>T p.G719C, c.2307_2308ins9 p.V769_D770insASV, c.2319_2320insCAC p.H773_V774insH, and c.2310_2311insGGT p.D770_N771insG. TaqMan EGFR Exon 19 Deletions Assay was designed to detect 19 deletions in EGFR exon 19. The TaqMan Mutation Detection Reference Assay was designed to a mutation-free region of the gene.

CastPCR reaction was run on a ViiA 7 real-time PCR system (Life Technologies) by incubating the samples at 95°C for 10 minutes, followed by 5 cycles of 92°C for 15 seconds and 58°C for 1 minute and then 40 cycles of 92°C for 15 seconds and 60°C for 1 minute.

The mutational status of a sample was determined by calculating the ΔCt value between amplification reactions for a mutant allele assay and gene reference assay, as follows. Normalized ΔCt = [Ct(mutant allele assay) – Ct(gene reference assay)] – calibration ΔCt. The calibration ΔCt value is the inherent Ct difference between a mutant allele assay and a gene reference assay. The cut-off ΔCt values were experimentally determined in this paper. If the ΔCt is ≤ ΔCt cut-off, the mutation is detected. If the ΔCt is > ΔCt cut-off, the mutation is not detected.

### 2.4. PCR Amplification and Direct Sequencing

PCR amplification and sequencing of genomic regions of the EGFR harbouring hot-spot mutations (exons 18, 19, 20, and 21) were performed as previously described [11]. PCR primers and conditions are available on request.

### 2.5. Length Analysis of Fluorescently Labelled PCR Products (Fragment Analysis)

Deletions in exon 19 were determined by fragment analysis after nested-PCR amplification with the use of a FAM-labelled primer [12]. Separation was done with a four-color laser-induced fluorescence capillary electrophoresis system (3500 DX Genetic Analyzer, Life Technologies). The collected data were evaluated with the GeneMapper 4.1v Analysis Software (Life Technologies).

### 2.6. Real-Time PCR (Allelic Discrimination) Assay

The L858R mutation on EGFR exon 21 was determined by an allelic discrimination real-time based approach, using specific primers and probes [12]. VIC-labelled probe was specific for the wild type sequence, whereas FAM -labelled probe was complementary to mutant. Runs were performed on a ViiA 7 real-time PCR system (Life Technologies). Sample ΔCt values were calculated as the difference between the mutation assay Ct (FAM-probe) and the wt assay Ct (VIC-probe) from the same sample. ΔCt values <2.5 indicate that the sample is mutant. PNA-clamp real-time PCR analysis for the L858R mutation was performed as previously described [12].

### 2.7. Therascreen EGFR RGQ PCR Kit

The Therascreen EGFR RGQ PCR kit (Qiagen) allows the detection of 29 somatic mutations in the EGFR oncogene by combining Scorpions and ARMS technologies. Samples were processed according to the manufacturer's protocol, using the Rotor-Gene Q real-time PCR cycler (Qiagen). The obtained data were analyzed with the Rotor-Gene Q Series Software (Qiagen).

## 3. Results

### 3.1. Identification of Thresholds and Analysis of the Training Set

A previous study suggested cut-off ΔCt values for EGFR TMDA [10]. However, a small number of FFPE NSCLC samples were assessed in this study (*n* = 22). Data analysis is significantly affected by the choice of the threshold value, and threshold Ct values should also be identified in order to limit the possibility of false positive results due to nonspecific PCR amplification. In order to identify the best threshold values for the EGFR TMDA, we first analysed with this method a training set of 30 NSCLC and 6 CRC samples, which were included as negative controls due to the rare presence of EGFR mutations in this tumor type. These samples had been previously evaluated for EGFR mutations by using routine diagnostic techniques including direct sequencing, fragment analysis, real-time PCR-allelic discrimination, and the EGFR Therascreen RGQ kit. The 6 CRC samples resulted to be wild type for EGFR mutations as expected (data not shown), whereas 14 NSCLC samples carried a mutant EGFR (Table 2).

**Figure 1:**
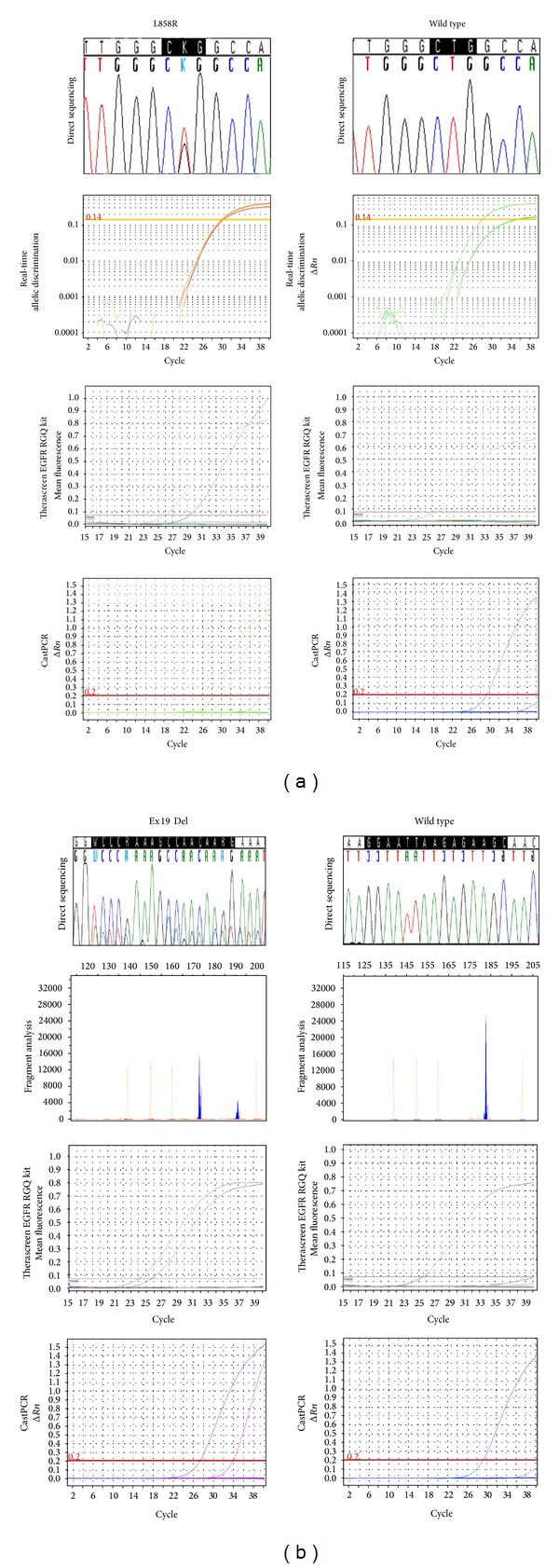
Representative results for EGFR mutation screening using direct sequencing, fragment analysis, real-time allelic discrimination, Therascreen EGFR RGQ kit, and EGFR TaqMan Mutation Detection Assays. (a) The right panel is an example of wild type EGFR, the left panel is an example of L858R mutation. (b) The right panel is an example of wild type EGFR, the left panel is an example of a deletion in exon 19 (c.2237_2254del18bp).

Threshold values allow determining the threshold cycle (Ct) for data analysis of amplification plots in real-time PCR assays. EGFR TMDA data were therefore analyzed by using several threshold values (0.2; 0.25; 0.3; 0.35) and results were compared with other methods for EGFR mutation detection. Our findings suggested that the specificity and sensitivity of castPCR technology were ensured by using for all employed EGFR mutation assays the threshold value of 0.2 for the analysis of study data. When using this parameter, samples with Ct ≤ 37 and/or a cut-off ΔCt value ≤7 were assessed as positive (Table 3). A 100% concordance between castPCR and routine diagnostic methods was observed in the 30 NSCLC samples of the training set, when using the above mentioned thresholds for data analysis (Table 2). In particular, castPCR was able to identify 14 EGFR mutations, including 8 exon 19 deletions, 5 L858R mutations, and 1 G719S mutation. Representative examples of data output using standard methods and castPCR are shown in Figure 1 for two cases with wild type EGFR and two cases carrying either the L858R mutation or an exon 19 deletion of the EGFR. The 6 CRC FFPE samples were confirmed to be negative for EGFR mutations by using the EGFR TMDA (data not shown).

### 3.2. Analysis of the Validation Set

We next analyzed by castPCR technology an additional subgroup of 28 NSCLC FFPE samples, including 14 EGFR wild type cases; 4 samples with L858R mutation; 1 with both L858R and T790M; 1 with G719A substitution; 1 with a L861Q mutation; 5 with exon 19 deletions; and 2 with exon 20 insertions. As shown in Table 2, castPCR did not show any false positive result in the wild type cases. However, only 10/14 EGFR mutations were identified by using this method. In particular, 2 insertions in exon 20 (p.D770-N771insNPH and p.D770-N771insY) and 2 deletions in exon 19 (p.E746_P753>VS and p.T751_I759>N) were identified by sequencing and fragment analysis, whereas castPCR failed to detect these mutations. These complex variants are rarely found in NSCLC and they are not included in the list of mutations detectable by the employed EGFR TMDA. Notably, such mutations were not detected even with the Therascreen kit.

One sample (2768) was wild type according to standard methods and showed the L858R point mutation by castPCR in two independent evaluations, whereas another sample (3000a) showed the L858R point mutation by Therascreen kit whereas castPCR was not able to identify the mutation in two different sets of analysis (Table 2). We analyzed these two samples with a real-time PCR-PNA clamp assay that confirmed the mutation in sample 2768 but not in sample 3000.

### 3.3. Comparison of Diagnostic Tests

Finally, we compared the mutation detection rate of sequencing, EGFR TMDA, and Therascreen in the analyzed 58 NSCLC samples. The three methods identified EGFR mutations with the same frequency although differences were observed between sequencing and real-time PCR based methods (Table 4). Sequencing could identify the rare exon 19 deletions and exon 20 insertions as described above but failed to detect common EGFR mutations (two exon 19 deletion and two L858R mutations) in samples with relatively low tumor content (samples 913a, 1262, 2376, and 3140). The length of exon 19 deletions was assessed by fragment analysis in these samples.

## 4. Discussion

Several studies have demonstrated that NSCLC patients carrying EGFR mutations significantly benefit from first-line therapy with specific TKIs [4]. Therefore, assessment of EGFR mutational status is mandatory in order to choose the most active treatment in NSCLC patients.

In this study, EGFR TMDA showed to be a robust method for mutational analysis since no reaction failure was observed in the analyzed samples. In addition, castPCR technology was highly specific and sensitive in detecting mutations in clinical samples from NSCLC patients. In particular, we identified threshold values for the use of castPCR by comparing this method with routine diagnostic techniques in a training set of samples. In this respect, the cut-off ΔCt values that we identified are different from what previously suggested by Didelot and collaborators [10] who defined cut-off values by analyzing 10 FFPE nontumor tissue samples. However, we screened a larger cohort of tumor samples, and we focused our attention on EGFR mutation-negative samples that were 22 in our training set (16 NSCLC and 6 CRC cases), whereas they were only 5 in the previous report. In addition, some assays differed between our study and the previous report on castPCR. In particular, we employed an exon 19 deletion assay that recognizes 19 different deletions. Finally, the more conservative thresholds that we propose allowed identifing known, common EGFR mutations in almost all the samples that we analysed with results that are very similar to the Therascreen RGQ EGFR kit that is widely used in clinical diagnostics.

Mutation testing techniques significantly differ for their limit of detection, as shown in Table 1. Although direct sequencing has a relatively low sensitivity, it is still considered the gold standard in clinical practice [3]. However, we found that sequencing failed to identify four mutations in samples with a relatively low tumor cell content that were detected by both castPCR and Therascreen. In contrast, a recent study reported that different EGFR mutation testing methods, including PCR-Invader, peptide nucleic acid-locked nucleic acid (PNA-LNA) PCR clamp, direct sequencing, Cycleave, and ARMS, were carried out comparably in the analysis of FFPE and cytology lung carcinoma samples [13]. The results of this latter study were significantly biased by the selection of the tumor samples. Indeed, the majority of FFPE samples selected by Goto et al. [13] had a tumor cell content of at least 50%. In Europe 50% to 70% of EGFR mutation analyses are performed on bronchial biopsy samples that usually contain a percentage of tumor cells that is lower than 50% [14]. Cytology samples often contain a low number of neoplastic cells and may also have significant amounts of nonneoplastic cells. Furthermore, increasing evidence suggests that even in tumors with EGFR mutations only a fraction of cells carry the mutant alleles that will be therefore diluted in a large background of wild type DNA [15]. In this regard, in the small cohort of biopsies analyzed in this study, 11/15 (73.3%) had a tumor cell content <50% and 3/4 samples for which sequencing produced a false negative result had <50% tumor cells. In addition, 6/14 (43%) of the cytology samples had <500 tumor cells. It is important to emphasize that PCR/sequencing might also lead to false positive results when analyzing small tumor samples, as recently suggested by the results of the Italian external quality assessment for EGFR mutations in lung cancer [16].

Sequencing has the advantage to identify novel and rare mutations that are not detected by targeted methods such as real-time PCR based assays, which can specifically identify known and predefined mutations. In this respect, EGFR TMDA failed to detect 2 insertions in exon 20 (p.D770-N771insNPH and p.D770-N771insY) and 2 deletions in exon 19 (p.E746_P753>VS and p.T751_I759>N) of the EGFR. However, probes used in the analysis were not specific for these mutations which are rarely represented in NSCLC (Catalogue of Somatic Mutations, www.sanger.ac.uk/genetics/CGP/COSMIC). Indeed, these mutations are not included in the list of mutations identified by the Therascreen EGFR kit, and we also failed to detect them using this method. Penzel and collaborators [17] have recently reported that 38% of the exon 19 deletions that they identified by sequencing in NSCLC samples were not included in the list of mutations identified by Therascreen. These data led the authors to conclude that the percentage of missed mutations is too high to recommend the use of mutation-specific PCR for diagnostic applications. We have recently revised a large number of samples screened for EGFR mutations for diagnostic purpose in our center (*n* = 800), and we found that 11.4% of samples carried deletions in exon 19 of the EGFR, with only 4 (0.5% of total cases analyzed) showing rare deletions not included in the list of mutations detected by either EGFR TMDA or the Therascreen kit (data not shown). Therefore, our data suggest that real-time PCR based methods can detect most of clinically relevant EGFR mutations in NSCLC.

The EGFR TMDA and the Therascreen kit detected the same number of mutations in our cohort of NSCLC samples. Only two cases both carrying an L858R mutation were discordant between these methods. By using a highly sensitive real-time PCR technique based on PNA clamping, we could confirm the mutation identified by castPCR but not the result of Therascreen. It is likely that mutant DNA is represented at a very low level in these samples and this might explain the different results that we obtained with these techniques. Nevertheless, the fact that PNA-clamp real-time PCR confirmed the L858R mutation in sample 2768 suggests that castPCR technology does not result in false positive findings. CastPCR is theoretically more sensitive as compared with ARMS technology (Table 1). However, the use of conservative cut-off ΔCt values might somehow limit the sensitivity of this assay. In this respect, it must be emphasized that extremely sensitive techniques might detect very low levels of mutant EGFR that are not associated with sensitivity to EGFR tyrosine kinase inhibitors. In particular, in the study by Zhou and collaborators a statistically significant difference was found between groups identified for high and low mutant EGFR content for progression free survival (11.3 versus 6.9 months, *P* = 0.014) and a trend for overall survival (15.9 versus 10.9 months, *P* = 0.062) [18]. Therefore, we feel that the parameters that we identified ensure an adequate balance between sensitivity and specificity of castPCR technology for the use in clinical samples.

## 5. Conclusions

Our data suggest that EGFR TaqMan Mutation Detection Assays powered by castPCR technology are a robust method that has shown an adequate sensitivity and specificity to detect clinically relevant EGFR mutations in samples from NSCLC patients.

## Figures and Tables

**Table 1:** Sensitivity of methods for mutational analysis.

Methods	Limit of detection*
PCR/sequencing	10–25
Fragment analysis	5
Real-time PCR (allelic discrimination)	Up to 5
ARMS (Therascreen)	Up to 1
castPCR	Up to 0.1

*Minimum percentage of mutant alleles in a wild type background required for reliable mutation detection.

**Table 2:** EGFR mutation detection in NSCLC samples.

Training set						Validation set					
Sample	Sample type	Tumor cells	Standard methods	CastPCR	Mutation	Sample	Sample type	Tumor cells	Standard methods	CastPCR	Mutation
731	FFPE (S)*	70%	∘	∘	WT	2826	FFPE (S)	80%	∘	∘	WT
732	FFPE (S)	50%	∘	∘	WT	2832	FFPE (S)	30%	∘	∘	WT
828	FFPE (S)	70%	•	•	Del15bp_ex19	2843	Cytology	300 cells	•	•	p.G719A
913a	FFPE (B)**	30%	•	•	p.L858R	2858	FFPE (S)	40%	•	•	p.L858R
966	FFPE (S)	70%	•	•	p.L858R	2903	FFPE (S)	30%	∘	∘	WT
1070b	FFPE (S)	90%	∘	∘	WT	2953	Cytology	1000 cells	•	•	Del9bp_ex19
1232a	FFPE (B)	60%	•	•	Del15bp_ex19	2958	FFPE (S)	90%	•	•	p.L858R-p.T790M
1262	FFPE (S)	40%	•	•	Del15bp_ex19	2965	FFPE (S)	40%	∘	∘	WT
1406	Cytology	1500 cells	∘	∘	WT	2971	Cytology	5000 cells	∘	∘	WT
1591	Cytology	>500 cells	•	•	p.G719S	2988	FFPE (S)	70%	∘	∘	WT
1674	FFPE (S)	50%	•	•	Del18bp_ex19	2992	FFPE (S)	80%	•	•	Del9bp_ex19
1677	FFPE (S)	60%	•	•	L858R	3023	FFPE (S)	60%	∘	∘	WT
2139	FFPE (S)	80%	∘	∘	WT	3031	FFPE (B)	20%	∘	∘	WT
2355	FFPE (S)	90%	∘	∘	WT	3032	FFPE (S)	20%	•	•	p.L861Q
2376	FFPE (B)	60%	•	•	Del12bp_ex19	3053	FFPE (B)	50%	∘	∘	WT
2572	Cytology	300 cells	•	•	Del15bp_ex19	3060	FFPE (S)	10%	∘	∘	WT
2659	Cytology	5000 cells	∘	∘	WT	3070	Cytology	150 cells	∘	∘	WT
2665	Cytology	250 cells	∘	∘	WT	3089	FFPE (B)	80%	∘	∘	WT
2693	Cytology	50%	∘	∘	WT	3098	FFPE (S)	70%	∘	∘	WT
2722	FFPE (S)	70%	•	•	Del15bp_ex19	3111	FFPE (B)	20%	∘	∘	WT
2733	Cytology	100 cells	∘	∘	WT	3140	FFPE (S)	20%	•	•	Del18bp_ex19
2739	FFPE (B)	40%	∘	∘	WT	3171	FFPE (S)	60%	•	•	p.L858R
2762	FFPE (B)	30%	•	•	Del15bp_ex19	3000a	FFPE (B)	5%	•	∘	Discordant p.L858R
2767	FFPE (B)	40%	•	•	p.L858R	2768	FFPE (B)	50%	∘	•	Discordant p.L858R
2778	Cytology	500 cells	∘	∘	WT	1672	Cytology	500 cells	•	∘	Discordant Del18bp_ex19
2787	FFPE (S)	80%	∘	∘	WT	2527	FFPE (S)	70%	•	∘	Discordant Del24bp_ex19
2789	FFPE (S)	80%	•	•	p.L858R	3014	FFPE (S)	80%	•	∘	Discordant ins9bp_ex20
2790	Cytology	250 cells	∘	∘	WT	3045	FFPE (S)	70%	•	∘	Discordant ins3bp_ex20
2798	FFPE (S)	90%	∘	∘	WT						
2824	FFPE (S)	80%	∘	∘	WT						

*FFPE tissue, surgical specimen; **FFPE tissue, small biopsy.

**Table 3:** Parameters for analysis of clinical samples with EGFR TaqMan Mutation Detection Assays powered by castPCR technology.

Parameter	Value
Threshold for data analysis	0.2
Ct threshold for mutant assays*	≤37
Cut-off ΔCt*	≤7

*Values required to assess a sample as positive.

**Table 4:** EGFR mutations detected by sequencing, castPCR technology, and Therascreen.

Mutation detected	Sequencing	CastPCR	Therascreen
Wild type	35	35	35
L858R	6	8	8
L858R + T790M	1	1	1
L861Q	1	1	1
G719A	1	1	1
G719S	1	1	1
EX19_DELETIONS	11	11	11
EX20_INSERTIONS	2	—	—

Total	58	58	58
